# The relationship between coping styles and career adaptability in telecommunications employees: chain mediation of anxiety and general self-efficacy

**DOI:** 10.3389/fpubh.2025.1664966

**Published:** 2025-09-19

**Authors:** Zhenyu Pan, Hanzhong Zhang, Xinqing Xu, Jingjing Song, Jinghua Zhu, Jiangang Shao, Yalei Li, Liping Jia

**Affiliations:** ^1^Department of Psychology, Shandong Second Medical University, Weifang, China; ^2^Department of Network Center, Shandong Second Medical University, Weifang, China; ^3^Department of Public Health, Shandong Second Medical University, Weifang, China

**Keywords:** telecommunication employees, coping styles, anxiety, general self-efficacy, career adaptability

## Abstract

**Objective:**

The rapid growth of the digital economy demands higher professional competence and adaptability from telecommunications employees. Therefore, it is particularly important to explore the influencing mechanism of career adaptability of telecommunications employees.

**Methods:**

A survey was conducted among 9,475 employees of telecommunication companies in 16 cities in Shandong Province using the Simple Coping Style Scale, Depression-Anxiety-Stress Scale, General Self-Efficacy Scale and Career Adaptability Scale. The collected data were processed and analyzed using SPSS 26.0. Mediation analysis was conducted using Hayes’ PROCESS macro (Model 6) with bootstrapping.

**Results:**

The results of correlation analysis showed that there was a significant correlation between coping style, anxiety, general self-efficacy and career adaptability of telecommunications employees (*p* < 0.01). Anxiety and general self-efficacy were identified as mediating factors between coping style and career adaptability, accounting for 4.522 and 28.334% of the total effect, respectively. At the same time, anxiety and general self-efficacy played a chain mediating effect between coping style and career adaptability, accounting for 12.059% of the total effect.

**Conclusion:**

This study found that coping style may directly affect career adaptability, and may also affect general self-efficacy through anxiety level, which in turn affects career adaptability. The results emphasize the importance of coping skills, emotion management and self-cognition, which is of great significance for improving the mental health level and career development ability of telecommunications employees.

## 1 Introduction

In recent years, the telecommunications industry has steadily developed into a key pillar of Digital China. The workplace environment is complex and changeable, the industry is changing rapidly, and the work intensity and pressure are huge, which puts higher demands on the quality of professional talents. There is an urgent need for telecommunications employees to have strong flexibility and adaptability (1). Career adaptability is the core of an individual’s entire career development. It refers to the psychological construction resources and processing capabilities that individuals have when facing current or future uncertain tasks and changes related to their professional roles. It can help individuals better solve various problems encountered in their career development (2–5). Lack of career adaptability will lead to an increased willingness of employees to leave, increase staff turnover, and further aggravate the shortage of talent in this field, affecting the healthy development of the industry (6). On the contrary, the improvement of career adaptability can enable employees to better adapt to and integrate into the telecommunications organizational culture, improve their job satisfaction (7, 8) and well-being (9), professional identity (10), work performance (11), etc., and prevent talent loss (12). Currently, the telecommunications industry is in a critical stage of digital transformation and rapid change. The industry environment is highly competitive and the work pressure is enormous. Telecommunications workers are a typical high-pressure occupational group. However, existing research on career adaptability mostly focuses on college students and young people (13–15), and there is a clear lack of attention to this specific group of telecommunications industry employees. Therefore, in the context of rapid changes and high pressure in the industry, it is necessary to conduct research on the mechanism of career adaptability of telecommunications employees, which will help prevent occupational risks in the telecommunications industry, alleviate occupational stress, and promote employee well-being and sustainable development of organizations.

Career construction theory suggests that coping style is an important factor in improving career adaptability. Coping style is the specific cognitive or behavioral approach that an individual adopts when facing difficulties, setbacks, and stressful events (16). It is an important factor affecting an individual’s environmental adaptability and mental health. Previous studies have found that positive coping style is positively correlated with career adaptability (17). According to career construction theory, if individuals can maintain a subjective positive response during their career development, they will be able to more effectively acquire adaptive resources and solve career problems, thereby promoting career development and success (18, 19). Previous studies have found that when faced with difficulties and setbacks, the more positive the coping style, the stronger the problem-solving ability. By proactively solving problems or seeking external support, individuals can better adapt to changes in the external environment, thereby continuously enhancing their adaptability (20). Therefore, coping strategies can positively predict career adaptability, but the mechanism between the two still needs further exploration.

When confronted with career uncertainty, the choice of coping styles often influence individual’s emotional state after dealing with stressful environments or events. When faced with stressful events such as job changes and personnel changes, employees may adopt negative coping strategies, which can easily lead to anxiety (21). Anxiety is excessive worry or fear experienced when an individual is unable to cope with or explain a threatening event. Negative coping styles often exacerbate these negative emotions. A study of medical professionals found that negative coping styles significantly increased anxiety levels and decreased life satisfaction (22). At the same time, anxiety can also reduce an individual’s career adaptability. Individuals with high levels of anxiety tend to have negative perceptions of future expectations and career development, which in turn reduces their career adaptability (23, 24). On the contrary, individuals who adopt positive coping strategies in stressful situations can more effectively manage stress, regulate negative emotions, and thus reduce anxiety levels (25). On this basis, individuals can narrow the gap between their actual state and their ideal state by regulating the intensity and direction of their emotional responses, and further develop career-related adaptive behaviors (26). Studies have found that career adaptability can be cultivated in teacher groups through effective emotional regulation (27, 28), and the lower the anxiety level, the higher the level of career adaptability (29). Therefore, coping style may affect an individual’s career adaptability by affecting his or her anxiety level.

In addition, general self-efficacy is another important factor affecting career adaptability (30). It refers to an individual’s prediction of whether he or she is capable of completing a specific task, which can enhance confidence in solving career-related problems (31, 32). From the perspective of social cognitive career theory, people are active and proactive, actively shaping their careers through beliefs and expectations. Only when people believe they can produce the desired outcomes through their actions will they be motivated to expend sufficient effort and resources to cope with difficulties and obstacles and ultimately achieve adaptive goals. Previous studies have found that the higher the level of general self-efficacy, the stronger the career adaptability individuals exhibit when facing career difficulties (33, 34). At the same time, studies have shown that individuals who adopt positive coping strategies in high-pressure situations tend to have higher general self-efficacy (35, 36). This makes them more inclined to actively solve problems, have stronger self-assessment abilities, and be able to make future plans. When faced with challenges and difficulties, they are often able to adapt to challenges more quickly and better, thus demonstrating higher levels of career adaptability. Therefore, coping styles may affect career adaptability through general self-efficacy.

Previous studies have also found a significant negative correlation between anxiety and general self-efficacy. Individuals with high anxiety have a more negative overall evaluation of their abilities (37). However, individuals with low anxiety tend to have optimistic expectations of their abilities and future outcomes, which enhances their general self-efficacy. When faced with various career developments and changes, individuals with low anxiety are able to adapt and adjust more quickly (38). In this process, active coping styles can reduce the anxiety caused by career uncertainty, help individuals maintain a positive attitude, enhance confidence in problem-solving, and improve general self-efficacy, thereby demonstrating greater adaptability in complex environments. Therefore, coping styles may jointly influence career adaptability through anxiety and general self-efficacy.

As described in the review, this study aims to explore the impact of coping styles on career adaptability, as well as the mediating role of anxiety and general self-efficacy between the two. By changing coping styles, reducing anxiety, and enhancing general self-efficacy, the individual’s career development ability is jointly shaped, thereby deepening the understanding of the formation mechanism of career adaptability and providing a scientific basis for the career development and mental health of employees in the telecommunications industry. Therefore, the following four hypotheses are proposed: H1: Coping style can positively predict career adaptability; H2: Anxiety plays a mediating role between coping style and career adaptability; H3: general self-efficacy plays a mediating role between coping style and career adaptability; H4: Anxiety and general self-efficacy play a chain mediating role between coping style and career adaptability.

## 2 Methods

### 2.1 Participants

The cross-sectional study was conducted from January to February 2024. We were commissioned by the Shandong Provincial Committee of the China Telecom Group Union to survey employees of Shandong Telecom. Employees were drawn from telecommunication companies located in 16 cities of Shandong Province. Stratified random sampling was employed to ensure representativeness. The sample size for each city was determined in proportion to its population share within the province. Employees from each city were randomly sampled. Online questionnaires were distributed to the selected employees, with the telecommunication companies in each city of Shandong assisting in forwarding the surveys to ensure that all respondents were from the telecommunication industry. A total of 10,335 online questionnaires were distributed through the “psycloud.com.” To minimize the impact of social desirability bias, we ensured that all respondents’ answers were anonymous and strictly maintained the confidentiality of the data, allowing them to freely express their genuine opinions. Before conducting data analysis, we tested the statistical hypothesis and found that the data were in accordance with normal distribution. After removing outliers and excluding unanswered questions by Mahalanobis distance method, 9,475 valid questionnaires were retained. The effective recovery rate is 91.679%. Before starting the questionnaire, all participants were informed about its purpose and assured of the confidentiality of their personal information. All participants voluntarily took part in the questionnaire. The study was approved by the local medical ethics committee.

### 2.2 Measures

#### 2.2.1 Simplified coping style questionnaire

In this study, the Simple Coping Style Questionnaire (SCSQ) developed by Folkman and translated and culturally adapted by Xie Yaning was used for measurement (39). This psychometric instrument comprises 20 items distributed across two distinct dimensions: positive coping (12 items) and negative coping (8 items). Responses are recorded on a 4-point Likert-type scale, with the differential between positive and negative coping scores serving as an indicator of an individual’s predominant coping style. Coping style score = positive coping standard score (Z) – negative coping standard score (Z). A score greater than 0 indicated that individuals tended to respond positively under stress. Less than 0, individuals tended to have negative coping style. In the current study, the SCSQ demonstrated robust internal consistency, with an overall Cronbach’s alpha coefficient of 0.915. The subscales also exhibited strong reliability, with alpha coefficients of 0.936 and 0.875 for positive and negative coping dimensions, respectively.

#### 2.2.2 Depression-anxiety-stress scale

The Depression-Anxiety-Stress Self-rated Scale in Simplified Chinese (DASS-21), compiled by Lovibond, revised by Gong Xu was used for the measurement (40). This comprehensive measure consists of 21 items evenly distributed across three dimensions: depression, anxiety, and stress. Respondents rate their experiences on a 4-point Likert scale, with higher scores reflecting more severe symptoms in each domain. The DASS-21 demonstrated strong internal consistency in this study, achieving a Cronbach’s α of 0.953 overall. The subscales for depression, anxiety, and stress also showed excellent reliability, with α coefficients of 0.886, 0.871, and 0.861, respectively.

#### 2.2.3 General self-efficacy scale

The study utilized the General Self-Efficacy Scale (GSES) developed by Schwarzer and revised by Wang Caikang for Chinese participants (41, 42). This 10-item scale employs a 4-point Likert scale, with higher total scores indicating greater general self-efficacy. In this study, the GSES exhibited a Cronbach’s α of 0.940.

#### 2.2.4 Career adapt-abilities scale

The Chinese version (43) the Career Adapt-Abilities Scale (CAAS) (44) was used to measure career adaptability. This 24-item scale comprises four dimensions: career concern, career control, career curiosity, and career confidence, each measured with 6 items. Utilizing a 5-point Likert scale, higher total scores indicate greater career adaptability. In this study, the CAAS demonstrated a Cronbach’s α of 0.987, with subscale α coefficients of 0.954, 0.964, 0.968, and 0.974 for career concern, career control, career curiosity, and career confidence, respectively.

### 2.3 Data analysis

This study employed SPSS 26.0 for data preprocessing, descriptive statistical analysis, analysis of variance (ANOVA), and correlation analysis. PROCESS 3.3 was utilized to conduct mediation analysis and testing. We used descriptive statistics to examine demographic information. We used Pearson’s correlation coefficients to analyze the relationships among coping styles, anxiety, general self-efficacy, and career adaptability. Finally, Mediation analysis was performed using PROCESS macro (model 6) to test the hypothesized chain mediation effects. All statistical tests were two-tailed, and the significance level was set at α = 0.05. According to previous studies, the SPSS macro program prepared by Hayes (45) for testing mediation effects is widely used and accepted, and the method is also mature, so this study adopted this statistical method for analysis.

## 3 Results

### 3.1 Common method bias tests

As data were collected from the same participants using self-reported questionnaires, common method bias could be a concern. Harman’s single-factor test was conducted to assess the potential for common method bias (46). Results revealed seven factors with eigenvalues greater than 1, with the first factor explaining 37.535% of the total variance. This value falls below the critical threshold of 40%, indicating that common method bias is not a significant concern in this study (47).

### 3.2 Descriptive statistics and correlation analyses

Basic information is shown in Table 1. The difference test results showed that there were significant differences among the variables in different demographic variables, so these factors were controlled as covariates in subsequent analyses.

**Table 1:** Demographic information of participants (*n* = 9,475).

Variables	Categories	Frequency	Percentage (%)	Coping styles	Anxiety	General self-efficacy	Career adaptability
Gender	Male	5,204	54.923%	0.019	7.115	2.714	83.917
	Female	4,271	45.077%	0.076	6.856	2.532	76.222
*t*				−2.516^*^	1.859	13.134^***^	16.088^***^
Age	≤28	1,718	18.132%	0.003	6.270	2.666	83.596
	28–35	2,851	30.089%	−0.059	7.380	2.602	79.544
	35–45	3,839	40.517%	0.065	7.206	2.618	79.223
	>45	1,067	11.261%	0.315	6.405	2.708	82.206
*F*				31.201^***^	13.694^***^	8.383^***^	17.268^***^
Years of service	≤5	3,070	32.401%	0.046	6.267	2.68	82.891
	5–10	1,862	19.651%	−0.083	7.463	2.589	78.954
	10–15	2,156	22.755%	−0.039	7.758	2.564	77.64
	15–20	1,237	13.055%	0.145	7.171	2.618	79.746
	>20	1,150	12.137%	0.296	6.59	2.714	82.371
*F*				26.856^***^	19.409^***^	15.905^***^	20.271^***^
Education	Technical secondary school	829	8.749%	−0.052	6.979	2.649	79.484
	Senior high school	412	4.348%	0.008	6.641	2.666	81.422
	Junior college	3,663	38.660%	−0.045	7.254	2.595	78.584
	Bachelor’s degree	4,077	43.029%	0.098	7.044	2.646	81.310
	Master’s degree and above	494	5.214%	0.464	5.057	2.732	87.970
*F*				28.128^***^	11.901^***^	6.311^***^	20.512^***^
Labor relations	Labor system employees	2,452	25.879%	0.232	6.388	2.711	84.511
	Outsourced staff	7,023	74.121%	−0.021	7.211	2.604	79.030
*t*				−9.809^***^	5.202^***^	−6.756^***^	−10.350^***^

*P < 0.05, **P < 0.01, ***P < 0.001, the same below.

Pearson’s correlation analysis was used to examine the relationships among coping styles, anxiety, general self-efficacy, and career adaptability of employees in the telecommunications industry. As shown in Table 2, coping styles were significantly positively correlated with general self-efficacy, career adaptability and its dimensions (*p* < 0.01), and significantly negatively correlated with anxiety (*p* < 0.01). General self-efficacy was significantly positively correlated with career adaptability and its dimensions (*p* < 0.01), and significantly negatively correlated with anxiety (*p* < 0.01). Anxiety was significantly negatively correlated with career adaptability and its dimensions (*p* < 0.01).

**Table 2:** Correlation between coping styles, anxiety, general self-efficacy, and career adaptability.

Variables	Scale score (Mean ± SD)	1	2	3	4
1. Coping styles	0.04 ± 1.10	–			
2. General self-efficacy	2.63 ± 0.67	0.264^**^	–		
3. Anxiety	6.99 ± 6.75	−0.452^**^	−0.259^**^	–	
4. Career adaptability	80.45 ± 23.48	0.388^**^	0.676^**^	−0.291^**^	–

*P < 0.05, **P < 0.01, ***P < 0.001, the same below.

### 3.3 Mediating effect

**Figure 1:**
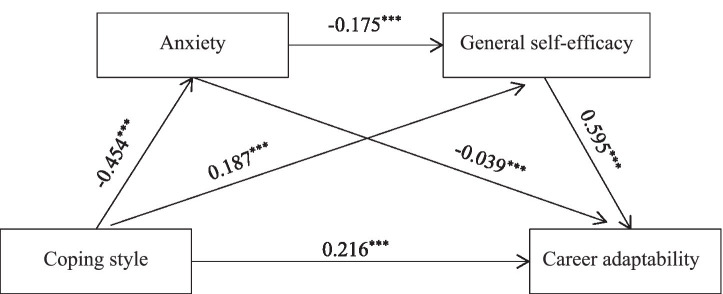
Diagram of the mediating effect model. ***p < 0.001.

The mediating effects of anxiety and general self-efficacy on the relationship between coping styles and career adaptability were examined using Model 6 in PROCESS with 5,000 bootstrap resamples to calculate 95% confidence intervals. As shown in Table 3, with career adaptability as the dependent variable and coping style as the independent variable, coping style positively predicted career adaptability (β = 0.391, *p* < 0.001). After adding anxiety and general self-efficacy as mediators, coping style negatively predicted anxiety (β = −0.454, *p* < 0.001) and positively predicted general self-efficacy (β = 0.187, *p* < 0.001). Anxiety negatively predicted career adaptability (β = −0.039, *p* < 0.001), while general self-efficacy positively predicted career adaptability (β = 0.595, *p* < 0.001). Notably, the direct effect of coping style on career adaptability remained significant (β = 0.216, *p* < 0.001) after accounting for the mediators. The chain mediation model is illustrated in Figure 1.

**Table 3:** Regression analysis between variables.

Dependent variables	Independent variables	*R* ^2^	*F*	*t*	β
Anxiety		0.209	416.887^***^		
	Coping styles			−49.252^***^	−0.454
	Gender			−1.417	−0.013
	Labor relations			−2.629^**^	−0.026
	Age			0.124	0.002
	Years of service			4.539^***^	0.065
	Education			1.177	0.012
General self-efficacy		0.115	176.104^***^		
	Coping styles			17.051^***^	0.187
	Anxiety			−16.086^***^	−0.175
	Gender			−14.129^***^	−0.139
	Labor relations			2.741^**^	0.029
	Age			−0.260	−0.004
	Years of service			−0.667	−0.010
	Education			0.363	0.004
Career adaptability		0.518	1270.166^***^		
	Coping styles			26.290^***^	0.216
	Anxiety			−4.781^***^	−0.039
	General self-efficacy			78.363^***^	0.595
	Gender			−12.557^***^	−0.092
	Labor relations			3.180^**^	0.025
	Age			−5.163^***^	−0.057
	Years of service			−0.124	−0.001
	Education			3.382^***^	0.027
Career adaptability		0.189	367.032^***^		
	Coping styles			41.884^***^	0.391
	Gender			−18.361^***^	−0.173
	Labor relations			4.528^***^	0.046
	Age			−4.160^***^	−0.059
	Years of service			−1.151	−0.017
	Education			2.668^**^	0.027

The test results of the mediating effects are shown in Table 4. Anxiety and general self-efficacy levels mediate the relationship between coping styles and career adaptability, with a total indirect effect value of 0.176. This includes three paths: coping styles → anxiety → career adaptability, with an indirect effect of 0.018; coping styles → general self-efficacy → career adaptability, with an indirect effect of 0.111; coping styles → anxiety → general self-efficacy → career adaptability, with an indirect effect of 0.047. The indirect effect values of the three paths account for 4.522, 28.334, and 12.059% of the total effect, respectively. The 95% confidence intervals do not include 0, indicating that the indirect effects are all significant.

**Table 4:** Mediating effects of anxiety and general self-efficacy on the relationship between coping style and career adaptability.

Path	Effect	Bootstrap SE	Boot LLCI	Boot ULCI	Percentage (%)
Indirect effect	0.176	0.007	0.162	0.189	44.966%
Indirect effect 1	0.018	0.004	0.010	0.025	4.522%
Indirect effect 2	0.111	0.007	0.097	0.125	28.334%
Indirect effect 3	0.047	0.003	0.041	0.054	12.059%

Indirect effect 1: coping styles → anxiety → career adaptability; indirect effect 2: coping styles → general self-efficacy → career adaptability; indirect effect 3: coping styles → anxiety → general self-efficacy → career adaptability.

## 4 Discussion

This study expanded upon previous researches by proposing a chain mediation model to explain how different coping styles influence career adaptability. We posited that anxiety and general self-efficacy served as key links in this chain, bridging the relationship between coping style and career adaptability. Our findings confirmed that individuals with positive coping styles tend to demonstrate greater career adaptability. Importantly, we uncovered a pathway through which this relationship unfolds. Specifically, effective coping mechanisms appeared to reduce anxiety, which in turn bolstered general self-efficacy, ultimately fostering enhanced career adaptability. This suggested that coping style not only directly shaped individuals’ capacity to navigate career challenges but also indirectly influenced it by shaping their emotional state and self-beliefs.

The results of this study showed that coping styles could positively predict the career adaptability of telecommunication employees, verifying Hypothesis 1. Specifically, individuals who tended to adopt positive coping styles had higher career adaptability and were better able to adapt to changes in life and work, which was consistent with previous research findings (48). According to career construction theory, individuals achieved good adaptation and development through interacting with the environment by either conforming to the environment or adjusting themselves. Positive coping was a prerequisite for positive adaptation. It was only through the adoption of positive coping strategies that individuals could calmly navigate and resolve challenges in both their professional and personal lives (49). The telecommunication industry was characterized by rapid technological updates and fierce market competition. Employees needed to learn new knowledge and new technologies continuously to adapt to the accelerated work pace. These changes brought pressure and challenges to employees. Adopting positive coping styles (such as seeking help from others, changing negative perspectives) could help employees improve their problem-solving abilities, effectively resolve career predicaments, and enhance career adaptability. In contrast, negative coping styles (such as avoidance, self-denial, etc.) might have led employees to develop negative emotions toward the work environment, reduce their coping abilities, and make it difficult for them to adapt to changes in their work.

Coping style not only directly affected career adaptability, but also indirectly affected it through anxiety levels. According to the conservation of resources theory, when facing work stress and career changes, employees who adopt positive coping strategies were better able to conserve their personal resources (e.g., time, energy, and emotional well-being) (50). This resource conservation prevented emotional exhaustion, reduced anxiety, and fostered positive emotions. Positive emotions, in turn, broadened individuals’ attention and cognitive scope, encouraged them to actively seek support, access more emotional and informational resources, and ultimately adapt better to new work roles and environments, thereby maintaining stable career development (51). Telecommunication employees often encountered numerous customer inquiries, including complaints, which exposed them to a high volume of negative information and emotional strain. This, coupled with demanding performance targets, depleted employees’ personal resources, leaving them ill-equipped to effectively address challenges. In essence, positive coping styles mitigated anxiety and promoted positive emotions, which subsequently enhanced individuals’ capacity for career adaptability (52, 53). Conversely, high anxiety levels could lead to detrimental consequences such as emotional exhaustion, increased burnout, and decreased job satisfaction, significantly impacting employees’ work performance and well-being (54).

At the same time, general self-efficacy also played a mediating role in the relationship between coping style and career adaptability. According to general self-efficacy theory, individuals’ past successful coping experience was an important factor which influences the formation of general self-efficacy. Individuals who adopt positive coping strategies had higher general self-efficacy, which was consistent with previous research (36). Furthermore, according to the social cognitive career theory, individuals who were willing and able (i.e., have high general self-efficacy) to take action to cope with constant changes could better solve career problems and had higher levels of career adaptability. In contrast, individuals with lower general self-efficacy tended to view challenging tasks as threats, lack confidence in their ability to complete work tasks, and were more inclined to maintain the status quo, making fewer proactive adaptations to environmental changes, which was not conducive to the development of career adaptability (55, 56). Under the strict performance appraisal system in the telecommunications industry, employees were under tremendous performance pressure. Employees with high general self-efficacy were more likely to believe in their ability to achieve goals through positive communication and effective problem solving, even when faced with customer complaints and demanding sales targets. This belief helped them turn pressure into motivation, making them more willing to try new working methods, improve their career adaptability, and better complete their work tasks.

In addition, this study further confirmed Hypothesis 4 by revealing that anxiety and general self-efficacy played a chain mediating role in the relationship between coping styles and career adaptability. Specifically, coping styles could influence career adaptability through their impact on anxiety, which in turn affected general self-efficacy. According to stress and coping theory, individuals engaged in cognitive appraisal when confronted with stressful situations. Those who leaned toward positive coping styles were more likely to positively assess their resources and believe in their work abilities, effectively mitigating anxiety stemming from environmental changes (57, 58). Conversely, individuals prone to negative coping strategies tended to doubt themselves when facing challenges, leading to heightened anxiety. Simultaneously, the cognitive model of anxiety suggests that anxiety could trigger negative thought patterns (59, 60). As a result, individuals with high anxiety were more likely to magnify potential threats, focus on negative information while overlooking positive cues, dwell on past failures, and harbor anxieties about the future. This hindersedtheir ability to objectively evaluate their capabilities, ultimately diminishing general self-efficacy. Over time, this can diminish work performance and impede the development of career adaptability (61). With the rapid advancement of digital transformation, telecom employees faced constant technological updates and business changes, an environment that could easily lead to high levels of work pressure and uncertainty. At the same time, customer service demands became increasingly diverse and complex, requiring employees to maintain a high level of emotional stability and professionalism. In this context, proactive coping strategies could help employees alleviate the anxiety caused by industry changes and service demands, thereby maintaining a high level of self-efficacy, helping them maintain confidence in facing unexpected issues and work challenges, and ultimately fostering greater career adaptability in a rapidly changing organizational environment. Therefore, it was crucial for telecommunication employees to acknowledge the significant role of psychological factors when navigating career transitions. By adopting positive coping strategies, they could effectively manage anxiety, foster positive self-perceptions, and ultimately achieve their career goals more effectively.

Our research has significant theoretical implications, expanding upon career construction theory and further emphasizing coping styles as an individual’s behavioral approach to proactively exploring in a changing environment, enhancing career adaptability, and helping individuals navigate the uncertainties and challenges of career development. Furthermore, by incorporating anxiety and general self-efficacy, we expand on the influence of individual emotions and cognition, placing greater emphasis on subjective initiative. Coping styles, low anxiety, and general self-efficacy contribute to the development of career adaptability. Our research also has practical implications. Amidst rapid technological change, organizations should guide employees to proactively respond and enhance their career adaptability to better mitigate the health risks and challenges posed by digital development. First, telecommunications technology is updated and iterated rapidly, and employees need to constantly learn new knowledge and skills. Companies can consider providing digital stress management workshops specifically for technological stress to help employees develop strategies to actively respond to technological updates and digital work and establish correct coping methods. Secondly, customer service demands in the telecommunications industry are becoming increasingly diverse and complex, and employees are under great emotional pressure. Companies can implement a mentoring system to pair new employees with experienced employees. By sharing customer communication experience, senior employees can guide new employees to effectively relieve their emotions and thus reduce anxiety. Third, telecom employees often worry about not being able to meet targets and doubt their own abilities due to strict performance appraisals. Organizations can establish a structured positive feedback mechanism to provide timely constructive affirmation and recognition of employees’ efforts, enhance employee confidence, and thus improve general self-efficacy. Ultimately, this will improve employee adaptability, alleviate stress, and help telecom employees maintain competitiveness and psychological well-being amidst technological innovation and industrial transformation.

## 5 Study limitations and future research directions

The present study has some limitations that need to be improved in future research. Firstly, the study employed a cross-sectional research design, which cannot determine the causal relationships among the four variables. Future experimental and longitudinal studies may be needed to further explore the mechanisms of action among these variables and enhance the persuasiveness of the conclusions. Secondly, this study examined only the impact of individual factors on career development, which may have limited explanatory power. Future research could include organizational factors. For example, incorporating organizational-level factors such as corporate culture and leadership styles, and examining how they interact with individual factors to collectively influence employees’ career adaptability and professional development. Third, the data in this study were all from self-reports of the subjects, and the data collection might have been affected by social desirability bias. Future studies should use mixed-methods approaches (e.g., adding qualitative interviews or supervisor ratings) to triangulate findings. Finally, the study sample was drawn solely from Shandong Province, which may limit the representativeness of the sample. Future research could expand the scope to a national level to test the validity of the results.

## 6 Conclusion

In sum, this study constructed a chain mediating model to explore the relationship between coping strategies and career adaptability, as well as the role of anxiety and general self-efficacy in this relationship. It aimed to provide an effective framework for understanding the relationships among coping strategies, anxiety, general self-efficacy, and career adaptability for employees in enterprises. Our research suggests a possible pathway, by learning and applying positive and effective coping strategies, employees can better manage stress, alleviate anxiety, further change negative thinking patterns, cultivate general self-efficacy, and ultimately enhance their career adaptability. This provides a new perspective and entry point for career adaptation.

## Data Availability

The raw data supporting the conclusions of this article will be made available by the authors, without undue reservation.

## References

[1] Hu J. Interpretation of the fourteenth five-year plan for the development of the information and communication sector. Telecom World. (2021) 23:11–3. doi: 10.13571/j.cnki.cww.2021.23.004

[2] Guan P Li M. Career construction theory: connotation, framework and applications. Adv Psychol Sci. (2015) 23:2177. doi: 10.3724/SP.J.1042.2015.02177

[3] Savickas ML. Career adaptability: an integrative construct for life-span, life-space theory. Career Dev Q. (1997) 45:247–59. doi: 10.1002/j.2161-0045.1997.tb00469.x

[4] Savickas ML. Career construction In: Career choice and development, vol. 149 (2002). 14–38.

[5] Savickas ML. The theory and practice of career construction In: Career development and counseling: Putting theory and research to work, vol. 1 (2005). 42–70.

[6] Sun C Xing Y Wen Y Wan X Ding Y Cui Y. Association between career adaptability and turnover intention among nursing assistants: the mediating role of psychological capital. BMC Nurs. (2023) 22:29. doi: 10.1186/s12912-023-01187-y, PMID: 36732804 PMC9894670

[7] Chan SHJ Mai X. The relation of career adaptability to satisfaction and turnover intentions. J Vocat Behav. (2015) 89:130–9. doi: 10.1016/j.jvb.2015.05.005

[8] Tolentino LR Garcia PRJM Restubog SLD Bordia P Tang RL. Validation of the career adapt-abilities scale and an examination of a model of career adaptation in the Philippine context. J Vocat Behav. (2013) 83:410–8. doi: 10.1016/j.jvb.2013.06.013

[9] Ding Y Li J. Risk perception of coronavirus disease 2019 and career adaptability among college students: the mediating effect of Hope and sense of mastery. Front Psychol. (2023) 14:1210672. doi: 10.3389/fpsyg.2023.1210672, PMID: 37649684 PMC10464948

[10] Rudolph CW Lavigne KN Katz IM Zacher H. Linking dimensions of career adaptability to adaptation results: a Meta-analysis. J Vocat Behav. (2017) 102:151–73. doi: 10.1016/j.jvb.2017.06.003

[11] Shu XL. Corporate employee career resilience and relationship between career satisfaction and job performance. Chin J Ment Health. (2019) 33:83–5. doi: 10.3969/j.issn.10006729.2019.01.014

[12] Zhang H Jiang JX Zhong MH Yu C Pang QY Mao YL. Career adaptability of newly graduated nurses at an obstetrics and Gynaecology Hospital in China: a qualitative study. J Nurs Manag. (2022) 30:2046–53. doi: 10.1111/jonm.13661, PMID: 35506471

[13] Gregor MA Weigold IK Wolfe G Campbell-Halfaker D Martin-Fernandez J Pino HVGD. Positive predictors of career adaptability among diverse community college students. J Career Assess. (2021) 29:115–28. doi: 10.1177/1069072720932537

[14] Hu X He Y Ma D Zhao S Xiong H Wan G. Mediating model of college students’ proactive personality and career adaptability. Career Dev Q. (2021) 69:216–30. doi: 10.1002/cdq.12269

[15] Liang Y Zhou N Dou K Cao H Li J-B Wu Q. Career-related parental behaviors, adolescents’ consideration of future consequences, and career adaptability: a three-wave longitudinal study. J Couns Psychol. (2020) 67:208–21. doi: 10.1037/cou0000413, PMID: 32105127

[16] Zhang X Yu L Chen Y Fu Z Zhang F Li Z. Career adaptability and career coping styles among Chinese medicine specialty students during the Covid-19: the mediating role of career decision-making self-efficacy. Heliyon. (2024) 10:e34578. doi: 10.1016/j.heliyon.2024.e34578, PMID: 39157377 PMC11327504

[17] Lin Z. The influence mechanism underlying meaning in life on career adaptability among college students: a chain intermediary model. Front Psychol. (2024) 15:1292996. doi: 10.3389/fpsyg.2024.1292996, PMID: 38500644 PMC10944908

[18] Shao Y Xu Y Zhang L Xu C Li M. Chain mediation of positive coping style and career adaptability in relationship between resilience and sense of meaning in life among adolescents. Occup Health. (2021) 37:3406–14. doi: 10.13329/j.cnki.zyyjk.2021.0805

[19] Zhang Z Zhao S Wang S Shao Y Duan X. Impact of work-family support on nurses' mental health: the chain mediating role of career adaptability and positive coping styles. J Bengbu Med Coll. (2024) 49:649–53. doi: 10.13898/j.cnki.issn.1000-2200.2024.05.021

[20] Hajitabar Firouzjaee M Sheykholreslami A Talebi M Barq I. Impact of the meaning in life on students' school adjustment by mediating problem-focused coping and self-acceptance. J Educ Psychol Stud. (2019) 16:59–76. doi: 10.22111/jeps.2019.4944

[21] Wanberg CR Banas JT. Predictors and outcomes of openness to changes in a reorganizing workplace. J Appl Psychol. (2000) 85:132–42. doi: 10.1037/0021-9010.85.1.132, PMID: 10740964

[22] Zhu W Wei Y Meng X Li J. The mediation effects of coping style on the relationship between social support and anxiety in Chinese medical staff during Covid-19. BMC Health Serv Res. (2020) 20:1007. doi: 10.1186/s12913-020-05871-6, PMID: 33148229 PMC7609823

[23] Pouyaud J Vignoli E Dosnon O Lallemand N. Career adapt-abilities scale-France form: psychometric properties and relationships to anxiety and motivation. J Vocat Behav. (2012) 80:692–7. doi: 10.1016/j.jvb.2012.01.021

[24] Gungor Tavsanli N Nehir S. Could intern health care students control their emotions and make a career plan during the Covid-19 pandemic? Inquiry. (2023) 60:469580231173268. doi: 10.1177/00469580231173268, PMID: 37209063 PMC10201068

[25] Folkman S Lazarus RS. Coping as a mediator of emotion. J Pers Soc Psychol. (1988) 54:466–75. doi: 10.1037/0022-3514.54.3.466, PMID: 3361419

[26] Wang H Ruan Q Liu X Long H Guo Z Wang Y. Longitudinal development of career adaptability in pre-service teachers: the impact of internship experiences and emotion regulation strategies. Humanit Soc Sci Commun. (2025) 12:1–13. doi: 10.1057/s41599-025-04966-x, PMID: 39310270

[27] Lee A Jung E. University students’ career adaptability as a mediator between cognitive emotion regulation and career decision-making self-efficacy. Front Psychol. (2022) 13:896492. doi: 10.3389/fpsyg.2022.896492, PMID: 36275236 PMC9581253

[28] Urquijo I Extremera N Solabarrieta J. Connecting emotion regulation to career outcomes: do proactivity and job search self-efficacy mediate this link? Psychol Res Behav Manag. (2019) 12:1109–20. doi: 10.2147/PRBM.S220677, PMID: 31853205 PMC6916693

[29] Tolentino LR Garcia PRJM Lu VN Restubog SLD Bordia P Plewa C. Career adaptation: the relation of adaptability to goal orientation, proactive personality, and career optimism. J Vocat Behav. (2014) 84:39–48. doi: 10.1016/j.jvb.2013.11.004

[30] Hirschi A. Career decision making, stability, and actualization of career intentions: the case of entrepreneurial intentions. J Career Assess. (2013) 21:555–71. doi: 10.1177/1069072712475287

[31] Bartley DF Robitschek C. Career exploration: a multivariate analysis of predictors. J Vocat Behav. (2000) 56:63–81. doi: 10.1006/jvbe.1999.1708

[32] Bandura A. Social cognitive theory: an agentic perspective. Annu Rev Psychol. (2001) 52:1–26. doi: 10.1146/annurev.psych.52.1.1, PMID: 11148297

[33] Vashisht S Kaushal P Vashisht R. Emotional intelligence, personality variables and career adaptability: a systematic review and meta-analysis. Vision. (2023) 27:316–28. doi: 10.1177/0972262921989877

[34] Zhang Y. The relationship between university students’ perceived social support and career adaptability: the mediating role of general self-efficacy In: Heilongjiang Researches on Higher Education (2019). 84–8.

[35] Yu P Chen J Wang Y Wang Z. Relationship between craving and abstinence self-efficacy in heroin addicts: the role of coping skills. Chin J Clin Psychol. (2019) 27:937–53. doi: 10.16128/j.cnki.1005-3611.2019.05.017

[36] Yuan Y Wu M Wang Z Li Z. Family socioeconomic status and children’s general self-efficacy: chain mediation of parents’care and coping style. Chin J Clin Psychol. (2020) 28:1009–12. doi: 10.16128/j.cnki.1005-3611.2020.05.030

[37] Judge TA Erez A Bono JE Thoresen CJ. The core self-evaluations scale: development of a measure. Pers Psychol. (2003) 56:303–31. doi: 10.1111/j.1744-6570.2003.tb00152.x

[38] Panari C Tonelli M Mazzetti G. Emotion regulation and employability: the mediational role of ambition and a protean career among unemployed people. Sustainability. (2020) 12:12 (22). doi: 10.3390/su12229347, PMID: 40881048

[39] Xie Y. A preliminary study of the reliability and validity of the brief coping styles scale. Chin J Clin Psychol. (1998) 2:53–4. doi: 10.16128/j.cnki.10053611.1998.02.018

[40] Gong X Xie X Xu R Luo Y. Psychometric properties of the Chinese versions of Dass-21 in Chinese college students. Chin J Clin Psychol. (2010) 18:443–6. doi: 10.16128/j.cnki.1005-3611.2010.04.020

[41] Schwarzer R Mueller J Greenglass E. Assessment of perceived general self-efficacy on the internet: data collection in cyberspace. Anxiety Stress Coping. (1999) 12:145–61. doi: 10.1080/10615809908248327

[42] Wang C Hu ZF Liu Y. Evidences for reliability and validity of the Chinese version of general self-efficacy scale. Chin J Appl Psychol. (2001) 7:37–40. doi: 10.3969/j.issn.1006-6020.2001.01.007

[43] Hou Z Leung SA Li X Li X Xu H. Career adapt-abilities scale—China form: construction and initial validation. J Vocat Behav. (2012) 80:686–91. doi: 10.1016/j.jvb.2012.01.006

[44] Savickas ML Porfeli EJ. Career adapt-abilities scale: construction, reliability, and measurement equivalence across 13 countries. J Vocat Behav. (2012) 80:661–73. doi: 10.1016/j.jvb.2012.01.011

[45] Hayes AF (2012). Process: A versatile computational tool for observed variable mediation, moderation, and conditional process modeling. University of Kansas. Available online at: http://www.afhayes.com.

[46] Zhou H Long L. Statistical remedies for common method biases. Adv Psychol Sci. (2004) 12:942. doi: 10.3969/j.issn.1671-3710.2004.06.018

[47] Xiong H Zhang J Ye B Zhang X Sun P. Common method variance effects and the models of statistical approaches for controlling it. Adv Psychol Sci. (2012) 20:757. doi: 10.3724/SP.J.1042.2012.00757

[48] Gurvich C Thomas N Thomas EHX Hudaib A-R Sood L Fabiatos K. Coping styles and mental health in response to societal changes during the Covid-19 pandemic. Int J Soc Psychiatry. (2020) 67:540–9. doi: 10.1177/0020764020961790, PMID: 33016171

[49] Lent RW Brown SD. Social cognitive model of career self-management: toward a unifying view of adaptive career behavior across the life span. J Couns Psychol. (2013) 60:557–68. doi: 10.1037/a0033446, PMID: 23815631

[50] Hobfoll SE Halbesleben J Neveu J-P Westman M. Conservation of resources in the organizational context: the reality of resources and their consequences. Annu Rev Organ Psychol Organ Behav. (2018) 5:103–28. doi: 10.1146/annurev-orgpsych-032117-104640

[51] Xanthopoulou D Bakker AB Demerouti E Schaufeli WB. The role of personal resources in the job demands-resources model. Int J Stress Manag. (2007) 14:121–41. doi: 10.1037/1072-5245.14.2.121

[52] Shao R He P Ling B Tan L Xu L Hou Y. Prevalence of depression and anxiety and correlations between depression, anxiety, family functioning, social support and coping styles among Chinese medical students. BMC Psychol. (2020) 8:38. doi: 10.1186/s40359-020-00402-8, PMID: 32321593 PMC7178943

[53] Wilkins KG Santilli S Ferrari L Nota L Tracey TJG Soresi S. The relationship among positive emotional dispositions, career adaptability, and satisfaction in Italian high school students. J Vocat Behav. (2014) 85:329–38. doi: 10.1016/j.jvb.2014.08.004

[54] Failo A Beals-Erickson SE Venuti P. Coping strategies and emotional well-being in children with disease-related pain. J Child Health Care. (2017) 22:84–96. doi: 10.1177/1367493517749326, PMID: 29258354

[55] Chuang Y-T Huang T-H Lin S-Y Chen B-C. The influence of motivation, self-efficacy, and fear of failure on the career adaptability of vocational school students: moderated by meaning in life. Front Psychol. (2022) 13:13. doi: 10.3389/fpsyg.2022.958334, PMID: 36211846 PMC9534183

[56] Guan Y Deng H Sun J Wang Y Cai Z Ye L. Career adaptability, job search self-efficacy and outcomes: a three-wave investigation among Chinese university graduates. J Vocat Behav. (2013) 83:561–70. doi: 10.1016/j.jvb.2013.09.003

[57] Wang L Kang C Yin Z Su F. Psychological endurance, anxiety, and coping style among journalists engaged in emergency events: evidence from China. Iran J Public Health. (2019) 48:95–102. doi: 10.18502/ijph.v48i1.787 30847316 PMC6401570

[58] Zhao Y. Investigation on anxiety and coping style of college students during Covid-19 epidemic. Psychiatr Danub. (2021) 33:651–5. doi: 10.24869/psyd.2021.651, PMID: 34928925

[59] Kashdan TB Roberts JE. Social anxiety's impact on affect, curiosity, and social self-efficacy during a high self-focus social threat situation. Cogn Ther Res. (2004) 28:119–41. doi: 10.1023/B:COTR.0000016934.20981.68

[60] Stopa L Clark DM. Cognitive processes in social phobia. Behav Res Ther. (1993) 31:255–67. doi: 10.1016/0005-7967(93)90024-O, PMID: 8476400

[61] Restubog SLD Ocampo ACG Wang L. Taking control amidst the Chaos: emotion regulation during the Covid-19 pandemic. J Vocat Behav. (2020) 119:119. doi: 10.1016/j.jvb.2020.103440, PMID: 32390659 PMC7206430

